# Silver molybdate: an excellent optical limiting material under nanoregime for photonic device application

**DOI:** 10.1038/s41598-024-53690-0

**Published:** 2024-03-07

**Authors:** B. Binish, B. Lokesh, Yukesh Veer, Silda Peters, M. Abith, T. C. Sabari Girisun, K. Mani Rahulan

**Affiliations:** 1 Nanophotonics Research Laboratory, Department of Physics & Nanotechnology, SRM Institute of Science and Technology, Kattankulathur, Tamilnadu 603 203 India; 2 Department of Physics, Baby John Memorial Government College, chavara, kollam, India; 3 Department of Chemistry, SRM Institute of Science and Technology, Kattankulathur, Tamilnadu 603 203 India; 4 Nanophotonics Laboratory, Department of Physics, Bharathidasan University, Tiruchirappalli, 620 024 India

**Keywords:** Silver molybdate, Z-scan, Reverse saturable absorption, Optical limiting, Applied optics, Lasers, LEDs and light sources, Optical materials and structures

## Abstract

There is a mounting demand for nonlinear optical materials with superior optical limiting performance which has a noticeable impact on protecting the delicate optical components from laser-induced damage. Transition metal molybdates have garnered attention in the nonlinear optics field due to their outstanding optical and luminescent properties, which give rise to widespread applications in next-generation optoelectronics devices. The structural confirmation of the as prepared silver molybdate nanoparticles were made by XRD and Raman spectroscopy analysis. The linear optical properties and the band gap of the synthesized material were studied using UV–Visible and photoluminescence spectroscopy. SEM analysis revealed the pebble like morphology of the silver molybdate nanostructures. The nonlinear responses of the samples were studied using open aperture z-scan approach with Nd:YAG pulsed laser (532 nm, 9 ns, 10 Hz). The sample exhibits reverse saturable absorption pattern attributed to the two photon absorption (2PA) mechanism. The obtained OL threshold value is in the order of 10^12^ which is suitable for fabricating optical limiters in nano second pulsed laser regime.

## Introduction

Unique optical materials with ultrafast response time, high nonlinearity, and optical confinement have been developed and used in various applications such as data storage, frequency conversion, optical switching, electro-optics, and optical confinement^1–3^. As the use of high-intensity pulsed lasers increases dramatically, it becomes increasingly important to protect fragile optical components from laser damage. Two-photon absorption (2PA), excited state absorption (ESA), free carrier absorption (FCA), nonlinear refraction, thermal defocus, and stimulated scattering have received much research in this regard^4,5^. The main requirement for a good nonlinear optical material is to have high optical quality in the solid state with large and stable optical nonlinearities. A wide range of materials with various nonlinear optical processes contributing to optical confinement and nonlinear absorption are being investigated^6^. Current advances in optical technology require the ability to adjust light intensity in a predictable and predefined manner. Optical limiters are such materials which have the ability to control the input fluency of the laser light having great demand in the current era. Thus the developing and fabricating efficient optical limiter is a hot topic of research^7,8^. Higher-order nonlinear absorption processes have been proposed as a means of improving spatial resolution and light transmission in certain applications. Two, three- and four-photon absorption methods (3PA and 4PA) have thus been demonstrated for optical power-limiting and fluorescence imaging^9^. By carefully choosing the appropriate matrix elements, the physical parameters can be easily optimized and various applications identified. Reverse saturable absorption phenomena, including nonlinear scattering, two- or three-photon absorption, transient absorption, interband absorption, and free-carrier and excited-state absorption, are known to be active in these nanostructures^10^. However, according to some studies, many materials exhibit multiple nonlinear absorption processes simultaneously when excited with comparable laser pulses. We cannot rule out the idea that many processes in the same material work together to create important optical confinement features. Therefore, it is important to distinguish between nonlinear absorption effects and determine nonlinear absorption parameters for materials exhibiting multiple nonlinear absorption effects. The most commonly used approach to characterize nonlinear absorption is based on nonlinear transmission measurements developed by Sheik-Bahae et al. introduced and commonly known as the Z-scan technique. This approach has been widely used as an effective and convenient tool for determining the nonlinear absorption properties of various materials^11,12^. As a result, organic materials have limited thermal and mechanical stability and a large potential for damage when exposed to high-intensity lasers, thus the search for innovative and effective nonlinear optical confinement materials has continued, which includes a wide range of organic and inorganic materials. Inorganic materials are of interest to researchers due to their special optical and nonlinear properties and excellent thermal stability. As a result, inorganic transition metals with high thermal stability, and optical and nonlinear properties have recently attracted the attention^13–16^. Silver molybdate is of particular interest among transition metal molybdates due to its excellent mechanical, chemical, and optoelectronic properties^7,17^. Metal molybdates can be synthesized using various methods such as hydrothermal synthesis, solid phase synthesis, mechanical synthesis, and sol–gel method. Among these, the co-precipitation synthesis method has proven to be the most efficient and cost-effective method for producing metal molybdate^18,19^. Current studies report the nonlinear absorption and optical confinement performance of silver molybdate nanoparticles to our knowledge, but no one has published the optical confinement properties of silver molybdate [Ag_2_MoO_4_]. Here we present the nonlinear absorption and optical confinement properties of silver molybdate nanostructures synthesized by co-precipitation method and probed using Z-scan technique and nanosecond Q-switched[7 ns] pulsed Nd:YAG laser with a wavelength of 532 nm.

## Experimental section

### Materials and methodology

**Figure 1:**
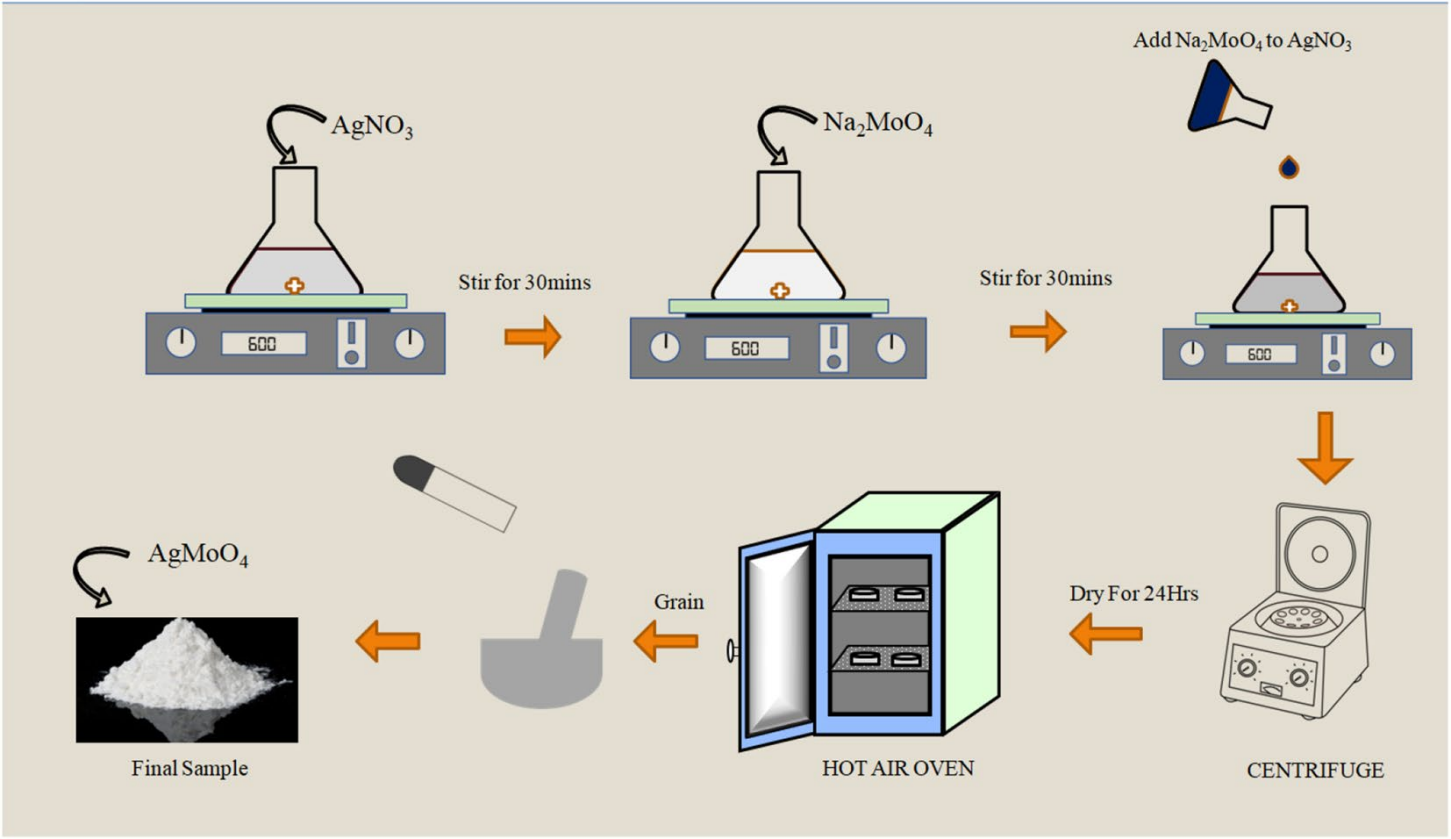
Schematic representation of synthesis of silver molybdate nanostructures.

Sodium Molybdate Dihydrate (Na_2_MoO_4_.2H_2_O) and Silver Nitrate Extrapure AR (AgNO_3_) was used as precursors. The precursors was purchased from SRL with analytical grade and used without further purification. Ag_2_MoO_4_ nanoparticles was synthesised by chemical precipitation technique. In a typical procedure 1.019 gm of silver Nitrate (AgNO_3_) and 1.451 gm of Sodium Molybdate Dihydrate was dissolved in 15 ml of deionised water separately under continuous stirring at 500 RPM for 30 min. Later Sodium Molybdate Dihydrate was introduced into silver nitrate solution under vigorous stirring. The chalky white precipitate is obtained upon the addition of Mo ions, the obtained precipitate is centrifuged at 6000 RPM for 5 min with ethanol until the PH becomes neutral. Followed by overnight drying at 60 °C and annealing at 600 °C for 4 h. The schematic representation of Ag_2_MoO_4_ synthesis procedure is depicted in Fig. 1.

### Material characterization

The structural characterization of Silver Molybdate (Ag_2_MoO_4_) nanostructures were analyzed by X-ray powder diffraction technique using Panalytical's X'Pert Pro instrument and Micro Raman spectroscopy was employed with the HORBA JOBIN Lab RAM HR instrument for further insights into the vibrational and lattice properties over a range of 100–1000 cm^−1^ range. The molecular vibrations and chemical bonding nature of the sample were identified by Fourier transform infrared spectroscopy using the SHIMADZU IRTRACER 100 instrument, covering a range of 400–4000 cm^−1^. High-resolution scanning electron microscopy (HRSEM) with the Thermo scientific Apreo S instrument was used to explore the nanostructures' morphological features, facilitating detailed analysis of their surface and shape and providing crucial insights into the chemical composition. The optical properties were investigated using ultraviolet–visible diffuse reflectance spectroscopy (UV–Vis DRS) on the Agilent Technologies Cary series 5000 instrument. X-ray photoelectron spectroscopy (XPS) utilizing the physical electronics technique was employed better to understand the oxidation states and composition of the nanostructures.

### Z-scan analysis

**Figure 2:**
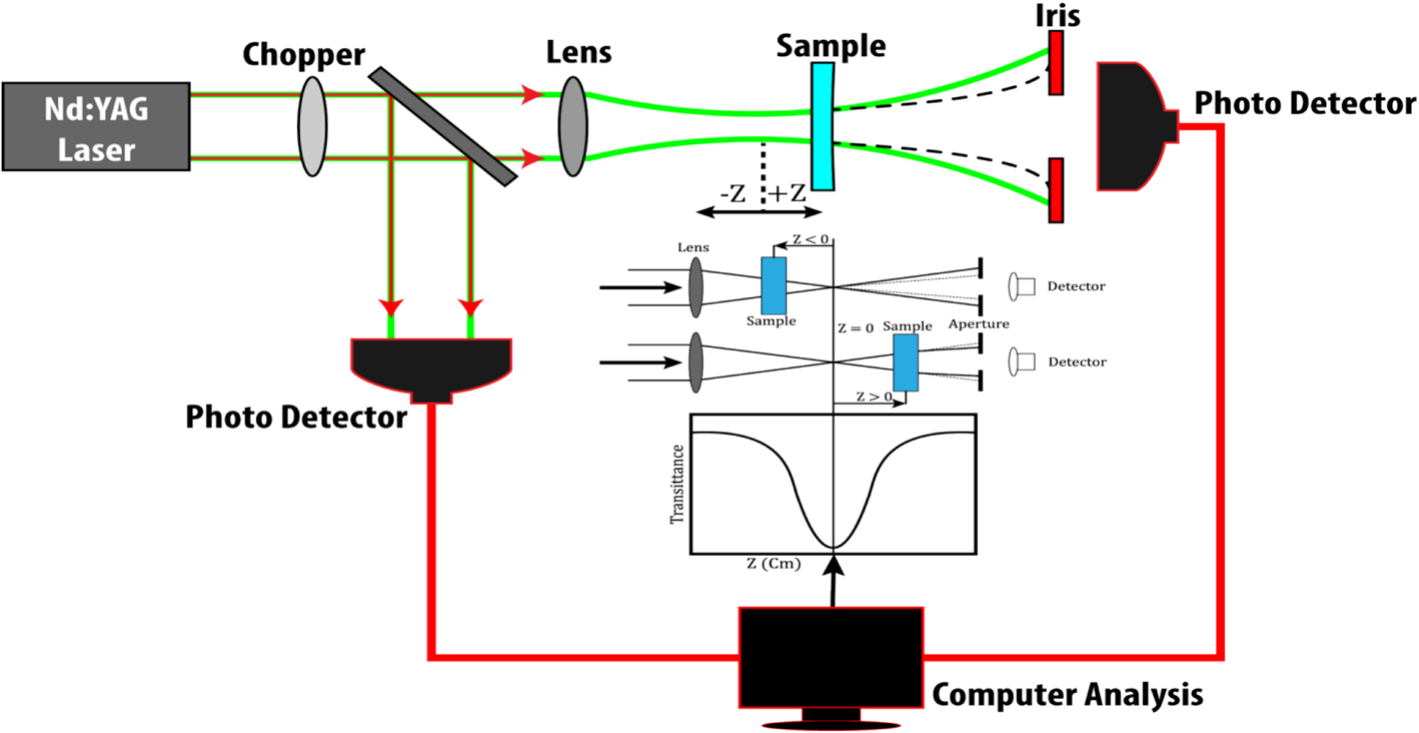
Schematic representation of Z-scan technique.

The investigation of nonlinear optical properties was carried out using the Z-scan technique, employing a Q-switched Pulsed Nd:YAG laser with a 5 ns pulse duration and a repetition rate of 10 Hz, generating a wavelength of 532 nm. A convex lens with a focal length of 15 cm was utilized to focus the laser beam onto the sample. The beam waist (ω0) at the focus was determined to be 16.9 μm, and the Rayleigh lengths (ZR) were calculated to be 35 mm and 7.42 mm. The experimental setup for the Z-scan technique is depicted in the Fig. 2.

### Computational methodology

DFT calculations were carried out in this study using the CP2K package with the exchange–correlation function set as generalized gradient approximation (GGA) parameterized by the Perdew-Burke-Ernzerhof (PBE) (GGA-PBE) functional^20,21^. The electronic wave function was described using the TZVP-MOLOPT-SR-GTH basis set^22^. Geometry optimization was performed using the BFGS optimization algorithm. With a convergence threshold of 1.0E-6. The crystal structure of Ag_2_MoO_4_was modeled and optimized. The optimized parameters are given in Table 1. The Quickstep method was utilized to calculate the total energy and electronic structure of the systems. Spin-polarized parametrization was taken into account during the calculations. The energy cut-off was set to 400 eV for all samples, and SFC tolerance and k-point were set to be 2 × 10^−6^ eV atom^−1^ and 1 × 2 × 1, respectively.

**Table 1:** Cell parameters obtained by DFT optimization of Ag_2_MoO_4_.

		Lattice size (Å)			Angle (°)		
		a	b	C	Α	β	Γ
Ag_2_MoO_4_	Simulated	9.3177	9.3177	9.3177	90	90	90

## Result and discussion

### X-ray diffraction analysis

**Figure 3:**
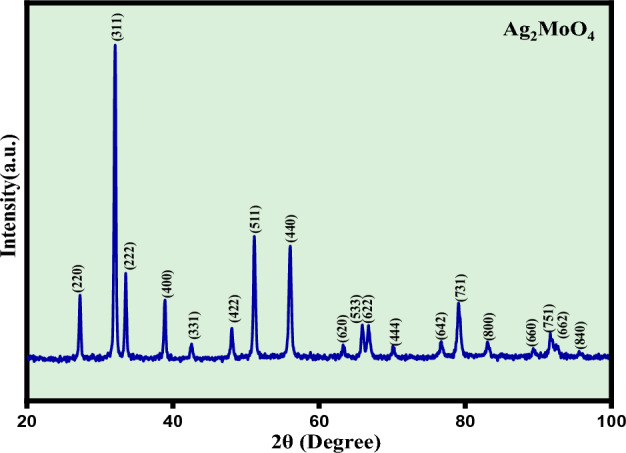
XRD spectra of Ag_2_MoO_4_ nanostructures.

The crystalline phase and lattice periodicity of the silver molybdate nanostructures were analysed by XRD analysis as depicted in Fig. 3. The diffraction peaks of the silver molybdate nanostructures confirm the cubic crystal system with Fd3m space group which is in consistent with the JCPDScard number. 08-0473^23–25^. The occurrence of the sharp and well defined peaks are the characteristics of structurally ordered nature of samples at long range and high crystallinity of the Ag_2_MoO_4_ nanostructures^26^. The average crystallite size was calculated used Deby Scherrer formula and found to be 79.1 nm respectively.

### Structural properties

**Figure 4:**
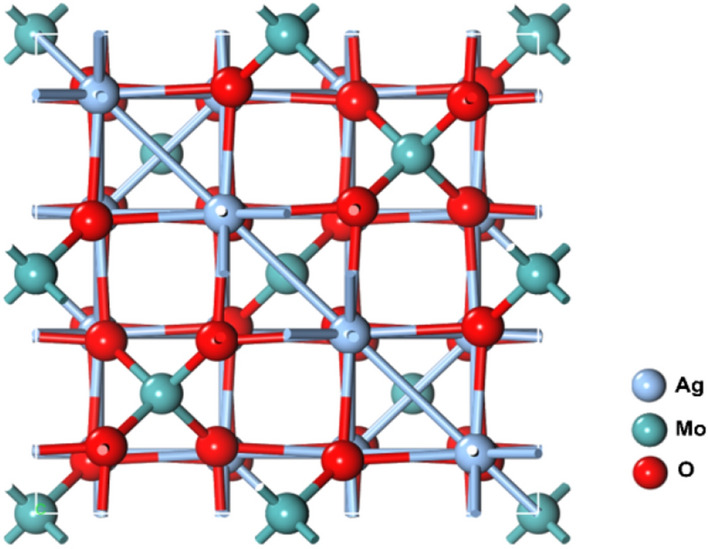
Crystal structure of cubic Ag_2_MoO_4_ nanostructures.

Ag_2_MoO_4_ belongs to the cubic geometry with space group Fd-3 m, similar to MgAl_2_O_4_. This phase diagram has been extensively studied both experimentally^27^ and theoretically^26^. Figure 4 displays the conventional cubic unit cell of Ag_2_MoO_4_, where Mo ions (cyan) occupy the tetrahedral 8a sites, while Ag ions (blue) reside at the octahedral 16d position. Oxygen atoms (red) stay at 32e positions.

The crystal structure of Ag_2_MoO_4_ is important for understanding its structural and electronic properties. The tetrahedral coordination of the Mo ions and octahedral coordination of the Ag ions lead to the hybridization of their d orbitals with the oxygen p orbitals, which plays a crucial role in determining the electronic properties of the material. The cubic structure of Ag_2_MoO_4_ also provides a high degree of symmetry, which allows for the efficient calculation of its electronic structure using various computational methods.

In summary, Ag_2_MoO_4_ has a cubic crystal geometry with space group Fd-3 m, similar to MgAl_2_O_4_. The crystal structure consists of Mo ions occupying the tetrahedral 8a sites, Ag ions residing at the octahedral 16d positions, and oxygen atoms at the 32e positions. The crystal structure of Ag_2_MoO_4_ plays a crucial role in determining its electronic and optical properties and provides a high degree of symmetry for efficient calculation of its electronic structure.

### Raman and FTIR spectroscopy

Raman spectroscopy was employed to further confirmation of the crystal structure's as well as the bonding type of the Silver molybdate (Ag_2_MoO_4_) nanostructures^28^. Group theory calculations show that molybdates with a cubic spinel structure have five Raman-active modes, which are described by Equation

1 $$\Gamma = {A}_{1g} + {E}_{g} + 3{T}_{2g}$$

**Figure 5:**
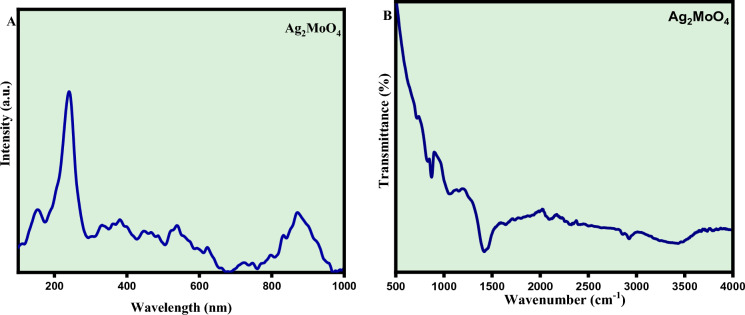
(**A**) Raman and (**B**) FTIR spectra of Ag_2_ MoO_4_ nanostructures.

Raman active modes are further classified as seven internal modes (owing to internal stretching and bending vibration) and six exterior modes (three rotation modes, two transitional modes, and undetermined modes)^29,30^. The A_1g_ Raman-active mode at 873 cm^−1^ attributes to the symmetric stretching vibration of the Mo–O bond in [MoO_4_] clusters. The T_2g_ mode found at 779 cm^−1^ corresponds to the asymmetric stretching vibration of Mo–O bond. The peak at 372 cm^−1^ in the tetrahedral [MoO_4_] cluster is a bending mode, whereas the E_g_ mode at 282 cm^−1^ reflects the lattice mode vibration of Ag cations. The T_2g_ modes at 90 cm^−1^, 352 cm^−1^ and 762 cm^−1^ are ascribed to the torsional vibrations of oxygen and molybdenum atoms within the tetrahedral clusters [MoO_4_]^31^. The structural vibrations in the octahedral clusters [AgO_6_] cause the E_g_ mode at 282 cm^−1^. Small variations in observed Raman peak position can be caused by various factors such as preparation methods, average crystal size, ion interaction forces, or the degree of structural order in the lattice. Moreover, active Raman modes reveal that Ag_2_MoO_4_ is structurally ordered at short distances^32,33^. The Raman spectrum of as-prepared Ag_2_MoO_4_ is shown in Fig. 5. A The peaks at 873 cm^−1^ are attributed to stretching modes of MoO_4_ units, the peak at 783 cm^−1^ to bridging Mo-O-Ag connections, the peaks in the 200–400 cm^−1^ range correspond to crystal bending modes. These findings are consistent with Gulbinski's. The symmetric and antisymmetric stretching vibrations of MoO_4_^2−^ cause four strong peaks in pure Ag_2_MoO_4_ at 763, 819, 873, and 995 cm^−1^ (R3). Internal and exterior vibrations of [MoO_4_]^2−^ tetrahedrons are classed as Raman active phonon modes of molybdate compounds^34^. Internal Raman modes are associated with vibrations within the [MoO_4_]^2−^ group with a fixed mass centre, whereas exterior Raman modes are associated with metal cation mobility with respect to the molecular [MoO_4_]^2−^ units (R5). The Raman mode estimated at 102 cm^−1^corresponds to triply degenerate F_2g_ associated with MoO_4_, T translations (MoO_4_). The symmetrically stretching vibration (Ag) of Mo–O bonds in [MoO_4_]^2−^ units is represented by the Raman signal at 865 cm^−1^, whereas the anti-symmetric stretching modes 3 (B_g_) and 3 (E_g_) of Mo–O bonds in [MoO_4_]^2−^ units are represented by the peaks at 821 and 760 cm^−1^ respectively^34^. It is an anti-symmetric mode with a large projection along the Mo–O bond in the cube diagonal direction. As a result, the peak at (802/865 cm^−1^ depends) which corresponds to an anti-symmetric mode with the MoO_4_ units A_1g_ symmetric stretching mode^34^. This extra peak is caused by the formation of deformed MoO_4_ tetrahedrons. This research confirms the existence of deformed MoO_4_ tetrahedron units in addition to conventional MoO_4_ tetrahedron units of scheelite type structure (R6). All of the peaks were identified and correspond to the stated values.

The FTIR spectra Fig. 5B, further confirmation of Ag_2_MoO_4_ formation. The absorption peaks at 3280 cm^−1^and 1650 cm^−1^ correspond to O–H stretching and bending vibrations of water molecules. A peak at 645 cm^−1^ confirmed the Ag–O stretching vibration of Ag_2_MoO_4_. The sharp peak at 891 cm^−1^ is assigned to the antisymmetric Mo–O stretching vibration of the tetrahedral MoO_4_^−2^ ion^24–35^.

### Optical studies

**Figure 6:**
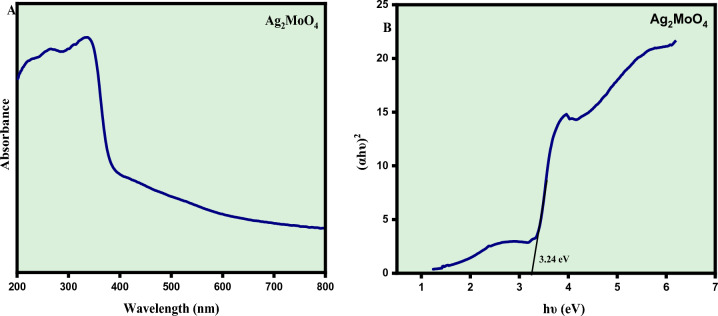
(**A**) UV–Vis absorption spectra and (**B**) Tauc Plot-Band gap of Ag_2_MoO_4_ nanostructures.

The optical properties of the Silver Molybdate (Ag_2_MoO_4_) nanostructures were investigated using UV–Vis absorption spectroscopy as shown in Fig. 6A. UV–visible absorption spectroscopy is a useful technique for monitoring the size-dependent optical properties of nonmaterial due to the photo generated electron–hole charge transfer within the material^37^. The Silver Molybdate (Ag_2_MoO_4_) nanostructures showed typical absorption in the Ultra-Violet region with maximum absorption between 250 and 400 nm. The broad UV range absorption can be directly explained by the charge carrier mechanism of the oxygen electrons in the 2p orbitals to (MoO_4_)^2−^ ions in the central Mo atom. The band gap of the Ag_2_MoO_4_was estimated by wood and Tauc plot using the relation

2 $${\alpha h\upsilon }={ A }{({h\nu }-{\mathrm{Eg}})}^{2}$$

where α, hυ, and Eg are the absorption coefficient, photon energy, and band gap respectively^32^. In this case the value calculated from the UV–Vis spectrum is 3.24 eV as shown in Fig. 6B. This result is consistent with the literature showing the existence of intermediate energy levels within the optical bandgap of Ag_2_MoO_4_ crystals. Therefore, they are formed by the structural disorder of tetrahedral [MoO_4_] and octahedral [AgO_6_] clusters, increasing the number of electron–hole pairs^38^.

### Electronic properties

Ag_2_MoO_4_ is a promising material with potential applications in solar energy conversion and other electronic devices. To understand its electronic properties, researchers have investigated the effect of pressure on its band structures. At ambient pressure of 1 bar, the calculated band structures of Ag_2_MoO_4_ have a band gap of 4.19 eV with an indirect transition from the valence band (VB) Γ point to the X point of the conduction band (CB) and a direct band gap at Γ of 4.40 eV. The bottom of the VB is mainly formed by hybridization between Ag 4d and O 2p orbitals, while the CB is mainly formed by hybridization between Mo 4d and O 2p orbitals. The d_x_^2^ − _y_^2^ and d_z_^2^ states of both transition metals produce the major contribution. The indirect and direct band gaps of Ag_2_MoO_4_ at 1 bar are 3.92 and 3.94 eV, respectively. An analysis of the projected density of states (DOS) shows that the VB maximum is derived mostly from O 2p and Ag d_z_^2^ orbitals with a minor contribution from dxy. The minimum CB is formed basically by Mo d_x_^2^ − _y_^2^ and d_x_.

**Figure 7:**
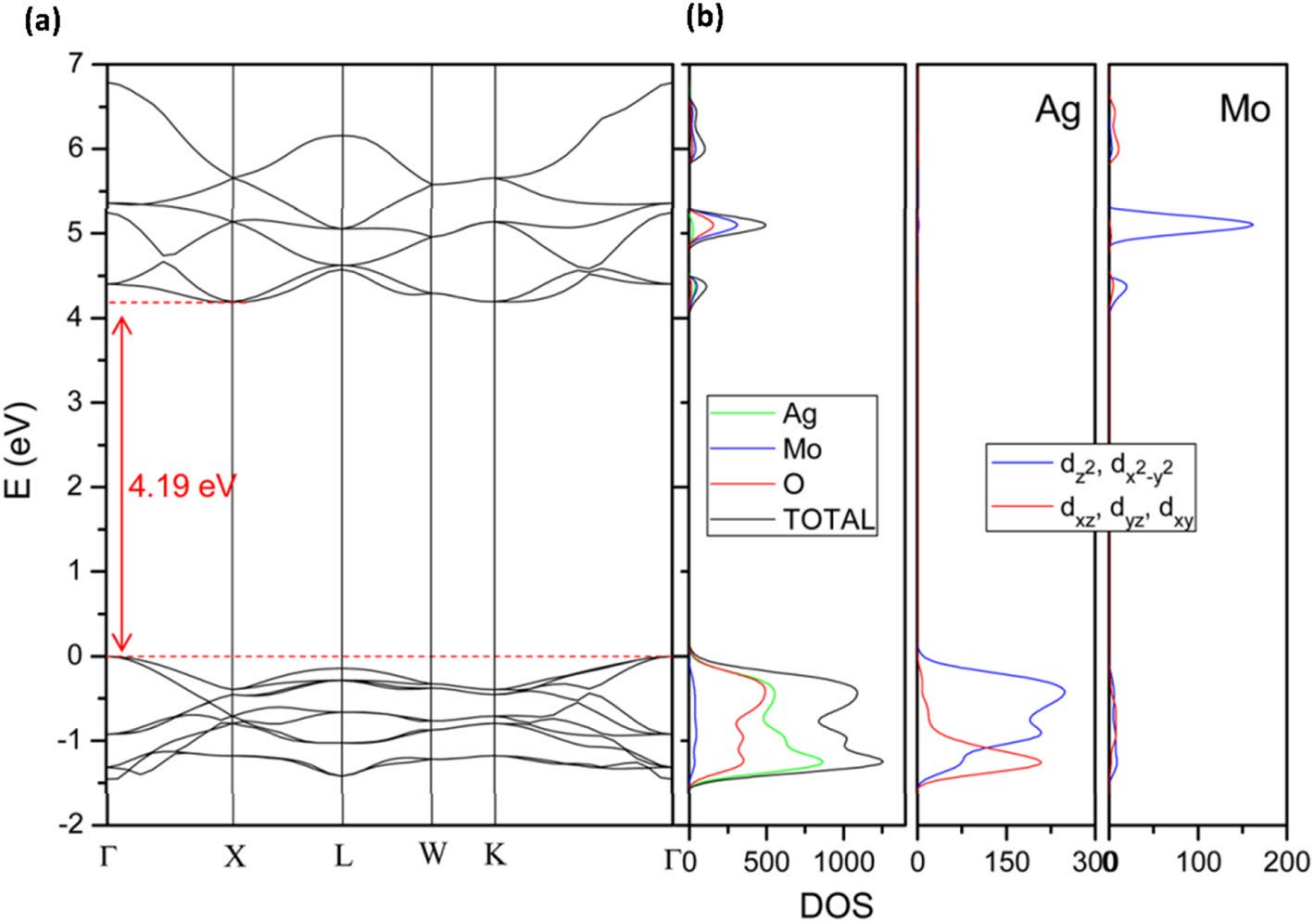
Band structure and DOS on atoms and orbitals for Ag_2_Mo_2_O_4_ at 1 bar.

Recently, Li et al. synthesized cube-like microstructures of Ag_2_MoO_4_ and deduced a band gap of 3.37 eV from their optical measurements^39^. This is lower than the calculated band gap at ambient pressure, which suggests that the band gap of Ag_2_MoO_4_can be tuned by changing its morphology. Furthermore, the projected DOS analysis shows that Fig. 7 the hybridization between different orbitals plays a crucial role in determining the electronic properties of Ag_2_MoO_4_. These results provide valuable insights into the electronic properties of Ag_2_MoO_4_ and can guide the design and optimization of electronic devices based on this material.

In summary, the effect of pressure on the band structures of Ag_2_MoO_4_ has been investigated, revealing an indirect and direct band gap of 3.92 and 3.94 eV, respectively, at 1 bar. The calculated band gap at ambient pressure is 4.19 eV with an indirect transition from the VB Γ point to the X point of the CB, and a direct band gap at Γ of 4.40 eV. The hybridization between Ag 4d and O 2p orbitals forms the bottom of the VB, while the CB is mainly formed by hybridization between Mo 4d and O 2p orbitals. The d_x_^2^ − _y_^2^ and d_z_^2^ states of both transition metals produce the major contribution. The results also suggest that the band gap of Ag_2_MoO_4_can be tuned by changing its morphology. These findings can provide important insights into the design and optimization of electronic devices based on Ag_2_MoO_4_.

### Morphology analysis (SEM)

**Figure 8:**
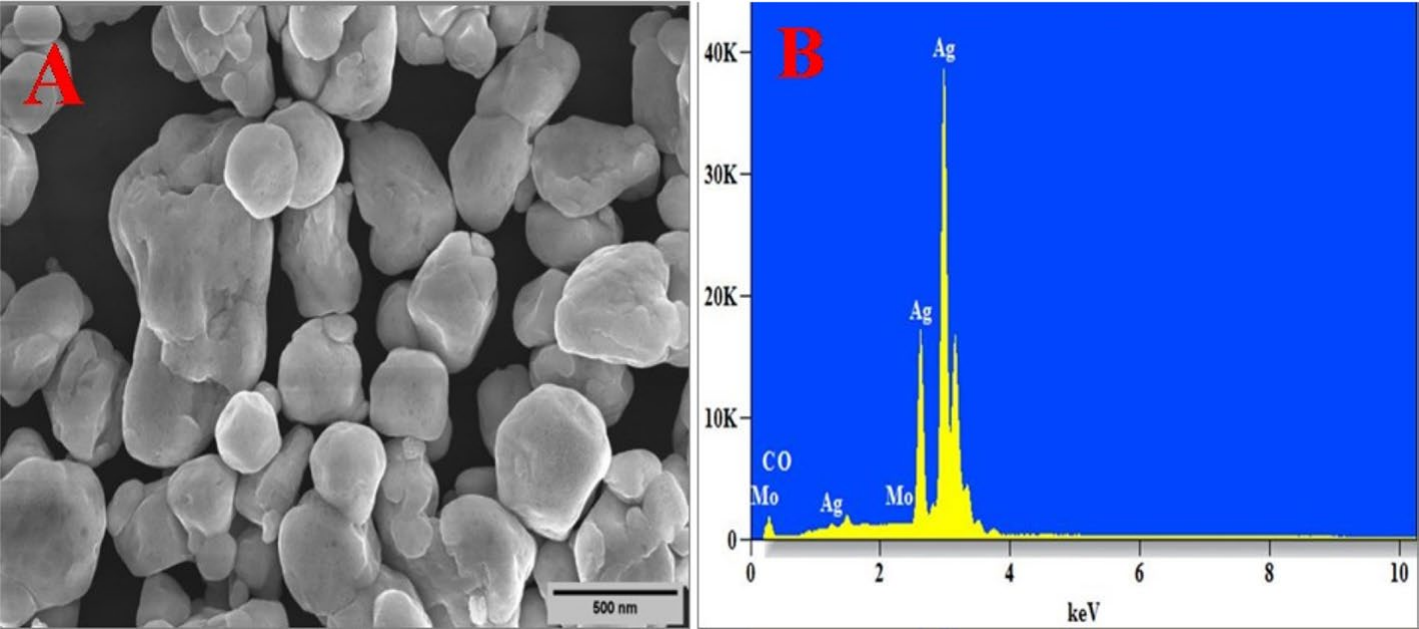
(**A**) SEM image and (**B**) EDAX Spectra of Ag_2_MoO_4_ nanostructures.

The surface morphology of the silver molybdate were analysed using Scanning Electron Microscopy (SEM) and elemental composition by energy dispersive X-Rayspectroscopy. SEM analysis exihibited pebble like morphology as depicted in Fig. 8A with an average particle size of 200 nm. Figure 8B shows the EDX spectra confirming the presence of the all the elemets in equimolar ratio and no additional elements were identified.

### X-ray photoelectron spectroscopy

**Figure 9:**
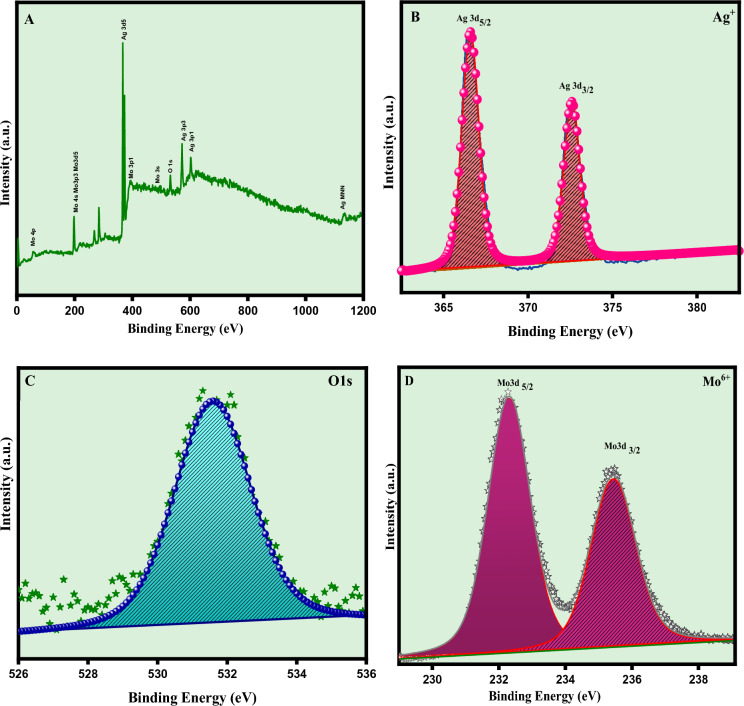
XPS spectra of Ag_2_ MoO_4_ nanostructures, (**A**) survey spectrum, (**B**) Silver 3d (**C**) Oxygen 1s and (**D**) Molybdenum 3d.

The surface elemental composition and the oxidation state of the as prepared (Ag_2_MoO_4_) silver molybdate nanostructures was analysed by X-ray photoelectron spectroscopy. Figure 9A depicts the high resolution survey spectra indicating the presence of silver, molybdate and oxygen. The obtained XPS peaks were calibrated by using the carbon 1s spectra at 285 eV. Figure 9B depicts the XPS spectra of Ag^+^ over a range of 364 to 381 eV having two peaks located at 366.6 eV corresponds to Ag 3d_3/2_ and the peak at 372.5 eV attributes to Ag 3d_5/2_ states confirming the 1 + state of silver^40^. The XPS spectra of molybdate over a range of 229 to 239 eV is shown in Fig. 9C, the obtained spectra is deconvoluted into two peaks with a splitting width of 3.1 eV. The peak obtained at 232.3 eV is ascribed to Mo 3d_5/2_ and the other peak at 235.4 eV corresponds to Mo 3d_3/2_ suggesting the + 6 oxidation state of Mo^41^. The oxygen 1s spectra consist of a broad peak located at 231.5 eV which is ascribed to the adsorbed O_2_ anions on the surface of the nanostructures as shown in Fig. 9D ^41^.

### Photoluminescence spectroscopy

**Figure 10:**
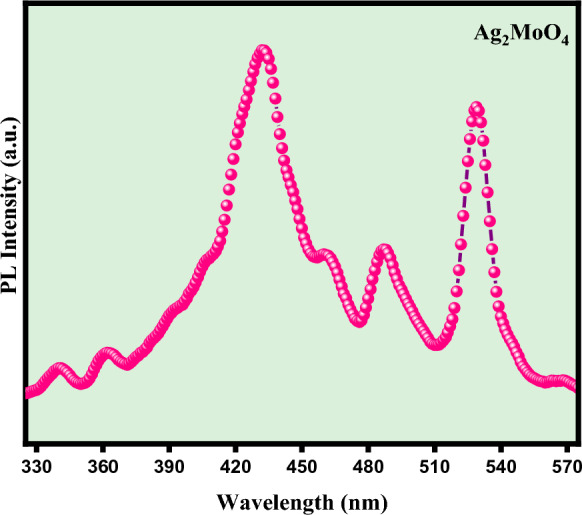
Pl spectra of Ag_2_MoO_4_ nanostructures.

Photoluminescence (PL) spectroscopy is crucial in interpreting the relationship between electron–hole pairs dynamics and spatial separation. The photoluminescence spectrum of Ag_2_MoO_4_nanostructures, as depicted in Fig. 10, exhibits a broad emission range spanning from 340 to 450 nm^42^. The PL profile signifies a distinctive multiphoton phenomenon wherein multiple energy levels or luminescent centres participate, entailing the entrapment of electrons within the band gap. This complex multiphoton process manifests due to interactions among various electronic states, leading to the intriguing spectral features observed in the photoluminescence spectrum^43^. The material's nonlinear properties can be substantially enhanced by minimizing these charge carriers' recombination rate. The observed photoluminescence spectrum reveals multiple energy levels or luminescent centers, contributing to a multiphoton process involving intricate electron trapping mechanisms within the material^44^.

According to Fabbro et al., the photoluminescence (PL) properties of Ag_2_MoO_4_ nanostructures are primarily influenced by distinct structural distortions within the material. These distortions arise from the differences between the crystal lattice's tetrahedral [MoO_4_] and octahedral [AgO_6_] clusters. The contrasting coordination environments of molybdenum and silver ions contribute to variations in the electronic band structure, which, in turn, impact the PL behaviour of the material^38^. Furthermore, changes in the bond lengths of Ag–O and Mo–O bonds significantly affect the photoluminescence characteristics of Ag_2_MoO_4_nanostructures. This variation in the bond lengths can lead to modifications in the electron distribution and local energy states within the crystal lattice. Consequently, this can influence the recombination dynamics of electron–hole pairs, which play a crucial role in determining the PL emission properties of the material. The intricate interplay between the Ag–O and Mo–O bond lengths contributes to the complexity of the PL spectrum and the observed multiphoton behaviour^45^.

In addition to the bond lengths, variations in the bond angles of O-Ag–O and O–Mo-O also contribute to the photoluminescence behaviour of Ag_2_MoO_4_nanostructures. The deviations from ideal bond angles in the crystal lattice introduce strain and local perturbations, creating multiple luminescent centres within the band gap. These luminescent centres act as trapping sites for charge carriers, affecting their mobility and recombination rates. As a result, the photoluminescence spectrum exhibits distinct features corresponding to the involvement of these multiple energy levels and electron trapping mechanisms, giving rise to the observed characteristic multiphoton process^46^.

The photoluminescence (PL) spectrum of Ag_2_MoO_4_ nanostructures has been analyzed, revealing the presence of three distinct peak emissions at 420, 460, and 480 nm (blue region). These emissions indicate specific distortions occurring in the Mo–O bonds within the [MoO_4_] clusters, significantly contributing to 9.48% of the total emission intensity in the spectrum. These peaks at different wavelengths suggest the involvement of various energy levels and electronic transitions associated with the specific Mo–O bond distortions, leading to discrete emission bands in the blue spectral region^47^.

A dominant peak exhibiting the highest emission intensity at 535 nm (green region) has been identified in the PL spectrum. This emission peak is closely associated with a high degree of structural organization and uniformity of the material contributing to 36.37% of the total emission intensity. The prominent emission at 535 nm reflects a well-defined electronic transition associated with a specific structural arrangement within the material, resulting in a broader emission band in the green part of the spectrum^48,49^. The investigation of Ag_2_MoO_4_nanostructures reveals interesting atomic configurations that lead to distortions in the [O–Ag–O] and [O–Mo–O] bonds within the [AgO_6_] and [MoO_4_] clusters, respectively^50,51^.

The observed distortions in the [O–Ag–O] and [O–Mo–O] bonds suggest that the local environments around the silver (Ag) and molybdenum (Mo) ions in the crystal lattice are not perfectly symmetrical. These distortions introduce strain and perturbations in the electronic band structure, leading to electron–hole pairs' different energy levels and transition pathways. As a result, when these charge carriers recombine, they emit photons at specific wavelengths, giving rise to the characteristic PL spectrum of Ag_2_MoO_4_^52^.

### Nonlinear optical studies

The nonlinear optical properties of the potential silver molybdate nano-system were examined by performing OA Z-scan measurement using nanosecond Q switched Nd:YAG laser with 532 nm laser excitation. To analyze the influence of irradiance on nonlinear absorption process, the experiment was performed at different input laser intensities over a range from 1.23 × 10^12^ W/m^2^ to 4.92 × 10^12^ W/m^2^ with a pulse width of 9 ns and repetition rate of 10 Hz. The silver molybdate nanoparticles were dispersed in diethylene glycol such that linear transmittance is 70%. Initially the experiment was performed for pure diethylene glycol and under the laser excitation in OA method, no significant absorption was observed which suggests the nonlinear absorption arises due to the silver molybdate samples. The sample exhibited reverse saturable absorption in open aperture z-scan mode for the silver molybdate nanostructures. The signature of reverse saturable absorption suggests that it can be explored as optical limiters. Since the bandgap of the material is around 3.2 eV which is higher than that of incident photon energy i.e., 2.33 eV, hence it not possible for an electron to have a direct transition from the valance band to the conduction band^28^. Therefore, with the given experimental condition multi photon absorption such as two photon or three photon absorption is the only possible mechanism for the transition of an electron from the VB to CB. So, for confirming the nature of MPA occurred in our sample the obtained experimental data were theoretically fitted for two and three photon absorption using the expression

3 $${T}_{OA}= \frac{1}{\begin{array}{c}{\left(1+\left(n-1\right)\beta {L}_{eff}{\left(\frac{{I}_{0}}{\left(1+{\left(\frac{Z}{{Z}_{0}}\right)}^{2}\right)}\right)}^{n-1}\right)}^{\frac{1}{n-1}}\\ \end{array}}$$

**Figure 11:**
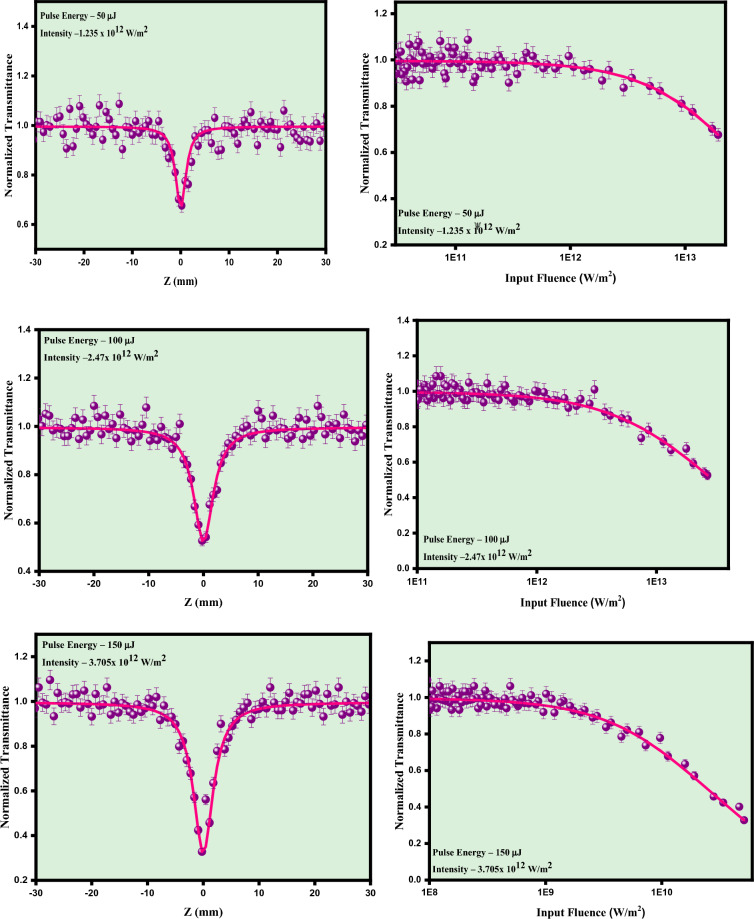

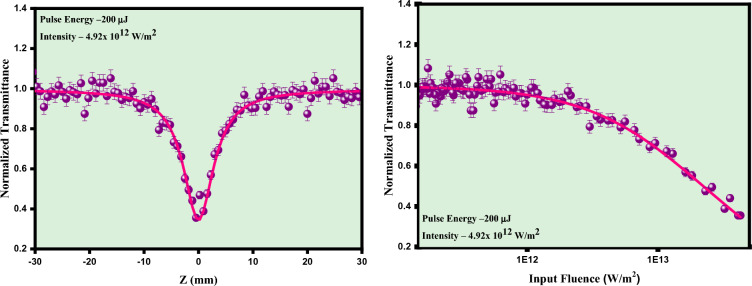
Intensity dependent open aperture and optical limiting traces of silver molybdate nanostructures.

The experimental data is best suited for 2PA equation and confirmed the nonlinear absorption originates from 2PA mechanism. The experiment is performed for different laser intensities over a range of 1.23 × 10^12^ W/m^2^ to 4.92 × 10^12^ W/m^2^ as depicted in Fig. 11 and the sample exhibited reverse saturable absorption for all the intensities and fitted well for the expression of 2PA. The intensity dependent study reveals that nonlinear absorption of the silver molybdate nanoparticles contingent on the input laser fluency. Based on the electron transition between intermediate states, the nonlinear two photon absorption can be occurred by means of two types, genuine and sequential 2PA. In genuine 2PA, the transition of the electrons to the excited state occurs by the absorption of two photons through the virtual states whereas in the sequential 2PA. the two photons sequentially absorbed and excite through real excited state^12^. The photoluminescence quantum yield measurements revealed that silver molybdate witnessed a quantum yield of 0.58% owing to two photon absorption process.

The intensity dependent OA Z-scan study reveals as the nonlinear absorption coefficient changes with the input fluency is the indication of sequential 2PA process, which is the nature of nonlinear absorption occurred within the sample. This 2PA can be explained by using energy level mechanism where the bandgap of the silver molybdate is 3.24 eV which is larger than compared to incident photon energy i.e. 2.33 eV. So, the domination of sequential 2PA happens because of the presence of near resonant states in the material under green pulsed laser excitation^18^. When the laser light irradiates the silver molybdate nanoparticles, initially excite an electron to the real excited state from the ground state and further due to the strong laser irradiation the electron sequentially absorbs a photon and transit to higher excited state. Since S_2_ being a virtual state the electron transits through phonon assisted non radiative process to the nearest electronic state thus the silver molybdate nanostructures exhibits sequential 2PA mechanism i.e. (1PA + ESA). Moreover the intensity dependent study as shown in Fig. 11 confirms the ESA as the NLA enhances with the increase in laser fluency. As the ESA leads to the depletion of ground state, the NLA will be varying with the input fluence resulting in variation in β values^53^. These findings confirm the involvement of sequential 2PA mechanism in the silver molybdate nanostructures. Moreover the Pl spectra in section “Photoluminescence spectroscopy” indicates the presence of distortions in the [AgO_6_] and [MoO_4_] clusters create defect sites and trap states within the band gap. These defect states can act as efficient electron and hole traps, reducing the recombination rate of electron–hole pairs and enhancing the nonlinear absorption of Ag_2_MoO_4_. Moreover, the variations in the local bonding environments can lead improved charge separation and mobility, further contributing to the NLA of the material^54,55^.

Using the open aperture data the OL parameters such as onset optical limiting and OL threshold can be calculated using the expression.

4 $${\text{F}}({\text{z}})=\frac{4\sqrt{{\ln }(2)}{{\mathrm{E}}}_{{\text{in}}}}{{\uppi }^\frac{3}{2}. {\upomega ({\text{z}})}^{2}}$$

The laser fluency dependent optical limiters are of great demand for restricting the input intensity of hazardous laser for protecting the sensitive sensors and human eyes. The variation of optical limiting response of the silver molybdate nanostructures under different laser fluency clearly elucidates that the strong nonlinear absorption due to the ESA make significant change in the optical limiting response of the sample^56,57^. The NLA (β) as well as the optical limiting threshold of the nanostructures is tabulated in Table 2. The obtained OL threshold is compared with recently reported materials in Table 3 making it a futuristic material for optical limiting devices. As the size of the nanoparticles decreases, the electronic band structure changes, leading to shifts in the absorption spectra and enhancement of nonlinear optical properties. In the present case, for silver molybdate nanoparticles, the average size measured is ~ 200 nm and profounds increase in optical cross-section enhancing the nonlinear absorption behaviour of the material. The presence of silver nanoparticles induces surface plasmon resonance and the induced plasmonic effects enhances the nonlinear absorption of silver molybdate. The SPR peak is observed towards the higher energies as the size of the nanoparticle is lower.

**Table 2:** Nonlinear absorption coefficient and optical limiting threshold of silver molybdate nanostructures.

Laser pulse energy (μJ)	Saturation intensity, Is × 10^11^ W/m^2^	Nonlinear absorption coefficient, β × 10^−10^ m/W	Optical limiting threshold, × 10^12^ W/m^2^
50	60	0.79	2.42
100	70	0.85	2.27
150	70	1.70	2.02
200	70	1.82	1.79

**Table 3:** Nonlinear response of recently reported materials.

Sample	Laser	NLO Response	β cm/W	Refs.
CdFe_2_O_4_	532 nm, 5 ns	2PA	0.29 × 10^−8^	^58^
MoS_2_	532 nm, 5 ns	RSA	0.75 × 10^−8^	^59^
CePO_4_	532 nm 5 ns	RSA	0.75 × 10^−10^	^60^
Cu_2_O	532 nm, 5 ns	2PA + ESA	6.4 × 10^−8^	^61^
Ag_2_MoO_4_ (present work)	532 nm, 5 ns	2PA	2.4 × 10^−8^	

## Conclusion

Silver Molybdate nanostructures were synthesized successfully by Co-Precipitation technique. XRD results confirmed the formation of crystalline and cubic phase of silver molybdate nanostructures, further confirmed by Raman and FTIR spectroscopy. The absorption spectroscopy reveled the absorption of sample in the visible region owing to charge transfer from O 2P orbital to Mo4d Orbital. The band gap of the sample were calculated to be 3.2 eV from absorption spectra using wood and tauc plot method. The nanostructured pebble like morphology of the sample were reveled by SEM imaging. The elements present and chemical oxidation state of the sample were identified XPS analysis. The optical limiting properties were identified by nanosecond pulsed Z-scan technique with a pulse excitation of 532 nm and pulse width of 7 ns. The sample exhibited reverse saturable absorption emerging from simultaneous absorption of two photons, the optical limiting properties of the sample were measured at different intensities and found to a excellent nanostructured material for fabricating optical limiters photonic devices.

## Data Availability

The datasets used and/or analysed during the current study available from the corresponding author on reasonable request.
